# Characterization of Anamnestic T-cell Responses Induced by Conventional Vaccines against Contagious Bovine Pleuropneumonia

**DOI:** 10.1371/journal.pone.0057509

**Published:** 2013-02-28

**Authors:** Philippe Totte, Aboubakar Yaya, Amadou Sery, Hezron Wesonga, Abel Wade, Jan Naessens, Mamadou Niang, François Thiaucourt

**Affiliations:** 1 Centre International de Recherche en Agronomie pour le Développement, UMR CMAEE, Montpellier, France; 2 Institut National de Recherche Agronomique, UMR1309 CMAEE, Montpellier, France; 3 Laboratoire National Vétérinaire, Garoua, Cameroon; 4 Laboratoire Central Vétérinaire, Bamako, Mali; 5 Kenya Agricultural Research Institute, Kikuyu, Kenya; 6 International Livestock Research Institute, Nairobi, Kenya; Federal University of Pelotas, Brazil

## Abstract

A better understanding of how T1 vaccination confers immunity would facilitate the rational design of improved vaccines against contagious bovine pleuropneumonia (CBPP). We show here that mycoplasmas-induced recall proliferation and IFN-γ responses are detected in cattle that received multiple shots of T1 vaccines. These anamnestic responses were under the strict control of CD4^+^ T lymphocytes. Moreover, CD62L expression indicated that both CD4^+^ effector memory (Tem) and central memory (Tcm) T lymphocytes are elicited in these animals. Comparative analysis with data from cattle that completely recovered from CBPP infection revealed similar anamnestic T-cell responses albeit at a lower magnitude for T1-vaccinated animals, particularly in the Tcm compartment. In conclusion, we discuss how our current understanding of T-cell responses will contribute to ongoing efforts for the improvement of future CBPP vaccines.

## Introduction

Improved vaccines are needed to control the spread of contagious bovine pleuropneumonia (CBPP), a devastating respiratory disease of cattle caused by *Mycoplasma mycoides* subsp. *mycoides* biotype Small Colony (*Mmm*SC), and a substantial economic burden for sub-Sahara Africa [1], [2], [3]. Indeed, the only vaccines currently in use are live vaccines, derived from the T1 strain and empirically attenuated through serial passage in embryonated eggs before production in artificial growth media [4]. Unfortunately these vaccines possess variable efficacy, induce short-lived immunity necessitating costly annual booster immunizations, and still retain some virulence causing sometimes adverse reactions at inoculation sites [5], [6]. Although some technical advances were accomplished in quality control and storage, recent efforts to address the limited efficacy of CBPP vaccines by increasing vaccine dosage and formulation resulted in little success [2], [7].

Comprehensive information on the nature of immunity induced by T1 vaccines will help improve their efficacy. Also, it may lead to the definition of predictors of vaccine efficacy that will allow a simple and rapid screening approach of novel vaccine formulations before fastidious and costly clinical trials in ruminants. As far as T1 vaccines are concerned, and apart from one report by Roberts et al in 1973 [8], research on immune responses triggered by vaccination has focused exclusively on humoral immunity. These studies indicate that antibody responses elicited by T1 vaccines are generally low and short-lived, and, thus, not appropriate to assess the immune status of animals or to serve as predictors of vaccine success [5], [9]. Preliminary work on cell-mediated immunity (CMI) failed to detect sensitization of lymphocytes in vaccinated animals but the methods used were of low sensitivity and specificity [8].

Here, we have analyzed T-cell immune responses induced by T1 vaccines using highly sensitive methods such as enzyme-linked immunospot (ELISPOT) for the detection of *Mmm*SC-specific IFN-γ production, and carboxyfluorescein diacetate succinimidyl ester (CFSE) to measure lymphocytes proliferation. The two currently approved vaccines, namely, T1/44 and its streptomycin-resistant derivate, T1sr, were used in this study. In addition, single immunization was compared to multiple annual booster immunization known to improve overall herd immunity. Also, we investigated the induction of both effector memory (Tem) and central memory (Tcm) T cells given their importance in maintaining long-term protective immunity [10]. Finally, a comparative analysis was performed between data from the present study and our knowledge of CMI induced in animals that recovered from CBPP.

## Materials and Methods

### 1. Ethics Statement

The African research institutions involved in the animal experiments did not have ethic committees operational at the time of the study. However, each received authorization to conduct the study from their respective ministry/government department namely the Ministry of livestock Development in Kenya, the Ministry of Livestock and Fisheries in Mali and the Ministry of Livestock, Fisheries and Animal Husbandry in Cameroon. In addition, all experiments were carried out according to the guidelines in the guide to the Care and Use of Experimental Animals provided by the French Ministry of Agriculture.

### 2. Animals and Vaccinations

Naïve zebu cattle above 3 years of age were purchased from CBPP-free areas in each country and were confirmed negative for antibodies to *Mmm*SC by ELISA. Animals were dewormed and treated against ticks when needed. They were confined in a paddock or a brick house, where they were fed on hay, water and mineral supplements. Vaccinations were performed as follow: i) at KARI in Kenya: animals received a single subcutaneous injection of a T1/44 vaccine batch; ii) at CVL in Mali, animals received two intramuscular injections at one month interval of a T1-SR vaccine batch; iii) at LANAVET in Cameroon, animals previously vaccinated twice on a yearly basis were purchased from a region were annual CBPP vaccination takes place under LANAVET’s supervision. At LANAVET, these animals received a third subcutaneous injection of a T1/44 vaccine batch. All vaccines preparations were quality controlled by the pan African veterinary vaccine centre (PANVAC) in Ethiopia and used at a dosage of 10^7^ viable mycoplasmas per injection [2]. Control nonvaccinated animals received PBS only and were kept with the vaccinated animals.

### 3. Proliferation Assays and Flow Cytometry

PBMC were isolated from peripheral blood as described previously [11] one month after the last vaccination and stored at −80°C in cryotubes containing 2–5×10^7^cells/ml in fetal calf serum supplemented with 10% DMSO until shipment to CIRAD under liquid nitrogen. Upon thawing, PBMC were loaded with CFSE (Invitrogen, France) at a final concentration of 1 µM before incubation for 9 days with 5 µg/ml of heat-inactivated *Mmm*SC (strain T1/44) or 2.5 µg/ml of the mitogen Concanavalin A (ConA) as a positive control and as described before [12]. Phenotype analysis of PBMC was performed as described previously with minor modifications [11], [12]. Briefly, cells were harvested and surface stained with the following primary monoclonal antibodies (mAb): mAb IL-A11 (IgG2a) for CD4, mAb BAQ92A (IgG1) for CD62L, mAb GC6A (IgM) for CD45R and mAb GC42A1 (IgG1) for CD45RO (all obtained from VMRD, Pullman, WA). Cells were then washed and stained with a cocktail of fluorochrome-conjugated, isotype-specific antibodies (Tebu, France). Debris and dead cells were excluded from analysis based on size and inclusion of 7-amino-actinomycin D (7-AAD) as a viability dye. Four-color analyses were performed with a FACScanto flow cytometer equipped with the FACSDiva ™ software (BD Biosciences) after the acquisition of at least 5,000 events. Control isotype antibodies were used to evaluate non-specific binding and to set gates and quadrants delineating positive populations. Proliferation levels are expressed as percentages of CFSElow cells which correspond to decreased CFSE fluorescence intensities relative to that in undivided cells. They measure the cumulated proliferation since day 0 irrespective of the number of division cycles.

### 4. IFN-γ Assays

The Bovigam™ ELISA (Prionics AG, Switzerland) was used to assess IFN-γ production in 5-days old supernatants of PBMC cultured as above. Briefly, supernatants were collected after centrifugation and stored at −20°C until assayed according to the manufacturer’s instructions; results are expressed as mean O.D. values of duplicates.

The bovine IFN-γ ELISpot^plus^ kit (Mabtech, Sweden) was used to establish the numbers of ex vivo antigen-responding cells in cultured PBMC and following the manufacturer’s instructions. Briefly, the assay was carried out in duplicates in polyvinylidene fluoride (PVDF) 96-well plates coated with a capture mAb specific for bovine IFN-γ. PBMC (2×10^5^/well) resuspended in RPMI-1640 supplemented with 10% FCS (Eurobio), 2 mM glutamine, antibiotics and 2-mercaptoethanol (Sigma) were stimulated with the same antigens as in the proliferation assay and incubated for 20 h at 37°C in a humidified atmosphere with 5% CO_2_. After extensive washing, the plates were incubated with a detection mAb against bovine IFN-γ. Finally, IFN-γ secreting cells or spots were stained using a combination of alkaline phosphatase (ALP), 5-Bromo-4-chloro-3-indolyl phosphate (BCIP) and nitroblue tetrazolium (NBT). Spot forming cells (SFC) were enumerated using an automated ELISPOT reader (Biosys, Germany). Results are presented as the mean number of *Mmm*SC-specific IFN-γ-spot forming cells/10^6^ PBMC.

### 5. Depletion Studies

Cells were depleted of CD4^+^ T cells by positive selection using a magnetic activated cell sorting system (Macs, Miltenyi Biotec, France) as previously described (12). Briefly, cells were stained with mAb ILA11 (IgG2a) against bovine CD4 (VMRD, USA) and incubated with Macs microbeads conjugated to goat anti-mouse IgG (H+L) prior to separation on MS columns and according to the manufacturer’s instructions. Unbound cell fractions collected from the columns repeatedly contained less than 5% CD4^+^ T cells as confirmed by flow cytometry.

### 6. Statistics

A nonparametric Mann-Whitney U-test (http://elegans.som.vcu.edu/~leon/stats/) was used to analyze differences between vaccinated and nonvaccinated animals. A difference was considered to be significant at a *P* value of <0.05.

## Results

### 1. Anamnestic Cellular Responses are Detected in Cattle that Received Multiple Shots of T1 Vaccines

**Figure 1:**
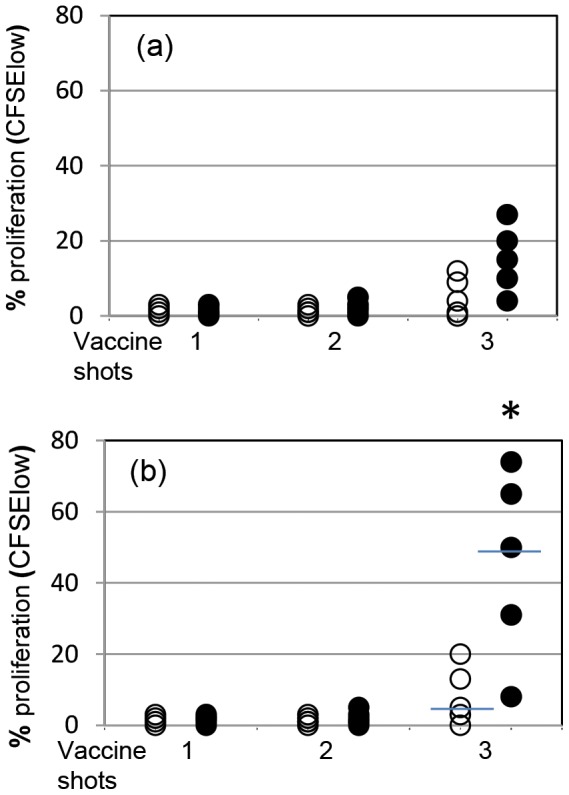
*Mmm*SC-induced recall proliferative responses of pbmc (a), and CD4^+^ T cells (b), collected from nonvaccinated (open circles) and vaccinated (closed circles) animals (n = 5) one month after single, double and triple vaccine inoculations. Results represent the net effect of *Mmm*SC stimulation (i.e., stimulated cultures minus non-stimulated cultures) and are representative of 2 independent experiments. Bars indicate median values and asterisks represent significant difference between vaccinated and naïve groups.

At individual levels, *Mmm*SC-specific recall proliferation in vaccinated cattle could only be detected in animals that received three shots of T1 vaccines (Fig. 1). However, at group levels, these vaccinated cattle could not be discriminated from nonvaccinated animals when recall proliferation of total PBMC was assessed (Fig. 1a). It is only when proliferation among CD4^+^ T lymphocytes was measured that significantly (*P* = 0.03) higher responses were detected in the vaccinated group versus the nonvaccinated group (Fig. 1b).

**Figure 2:**
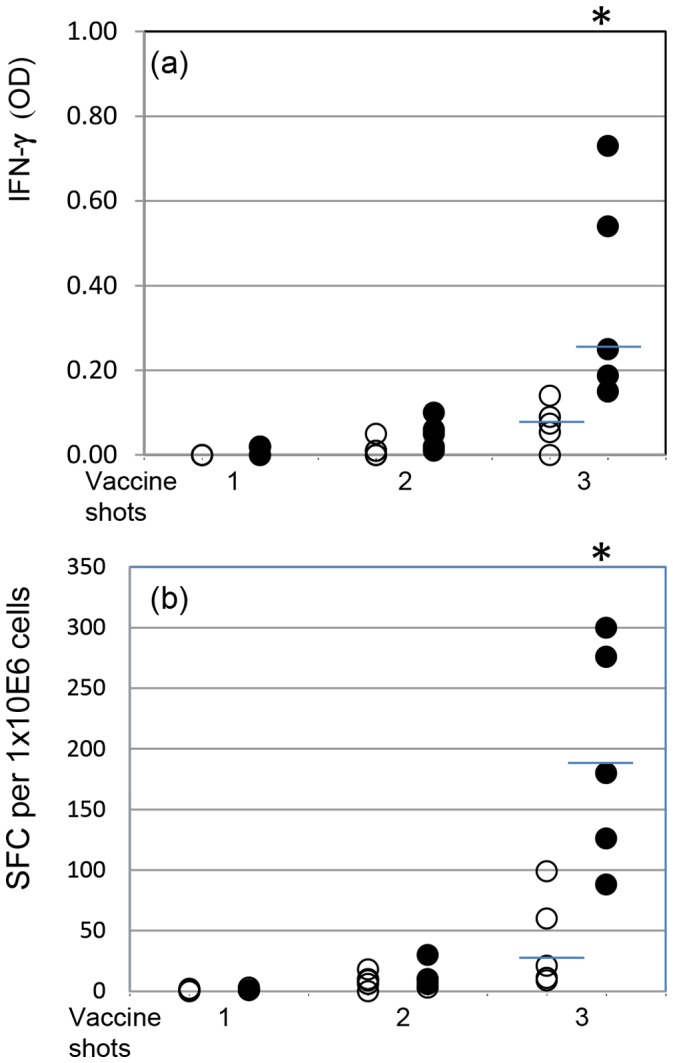
*Mmm*SC-induced recall IFN-γ responses measured by ELISA (a) and ELISPOT (b) using pbmc collected from nonvaccinated (open circles) and vaccinated (closed circles) animals (n = 5) one month after single, double and triple vaccine inoculations. Results represent the net effect of *Mmm*SC (i.e., stimulated cultures minus non-stimulated cultures) and are representative of two experiments. Bars indicate median values and asterisks represent significant difference between vaccinated and naïve groups.

*Mmm*SC-induced recall IFN-γ responses in vaccinated animals were also detected at substantial levels only in animals receiving multiple vaccine injections (Fig. 2). At group levels, both ELISA (*P* = 0.028) and ELISPOT (*P* = 0.016) methods allowed discrimination between vaccinated and nonvaccinated animals (Fig. 2a&b).

Recall proliferative and IFN-γ responses to the mitogen ConA were comparable for all groups irrespective of the vaccination status and number of vaccine shots (see Fig. S1).

### 2. Anamnestic Cellular Responses Induced by T1 Vaccines are under the Strict Control of CD4^+^ T cells

**Figure 3:**
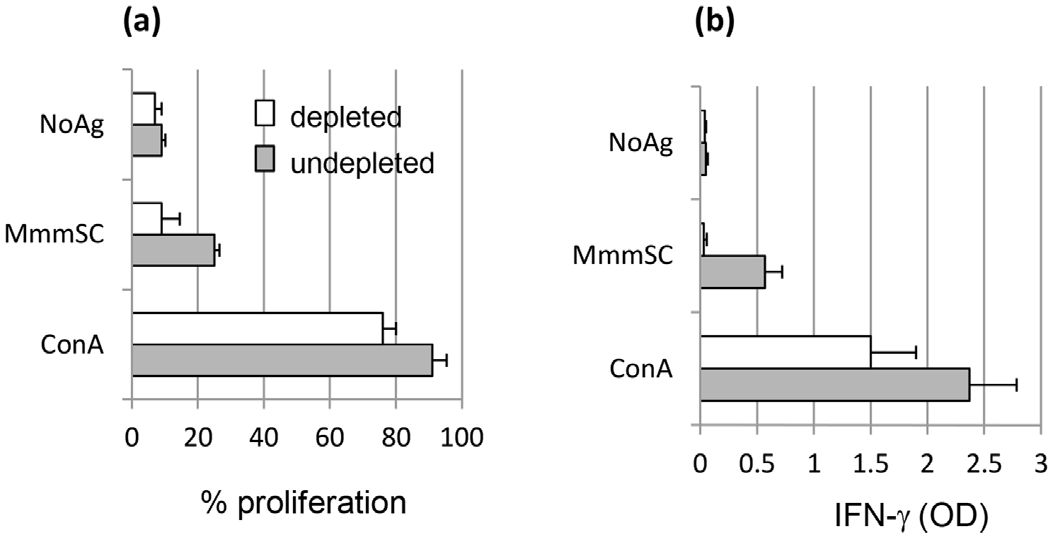
Effect of CD4 depletion on *Mmm*SC-induced recall proliferation (a) and IFN-γ (b) responses of pbmc collected from vaccinated animals. Cells were incubated in the absence (NoAg) or presence of *Mmm*SC Ags (MmmSC), or in the presence of the mitogen Concanavalin A (ConA) as a positive control. Results are expressed as mean percentages (± standard deviation) from three animals.

In vaccinated animals showing the strongest T-cell responses, *Mmm*SC-induced recall proliferation was evident among cells that did not belong to the CD4 sub-type although to a lesser extent than in the CD4^+^ population. Given the potential inhibitory properties of CD4^+^ T lymphocytes (i.e., Tregulatory or Treg) it was of interest to analyse immune responses occuring in their absence. Therefore, depletion studies were performed and clearly indicated that both proliferation and IFN-γ production induced by *Mmm*SC Ags required the presence of CD4^+^ T lymphocytes (Fig. 3).

### 3. Both CD4^+^ Tem and Tcm are Elicited by T1 Vaccines

**Figure 4:**
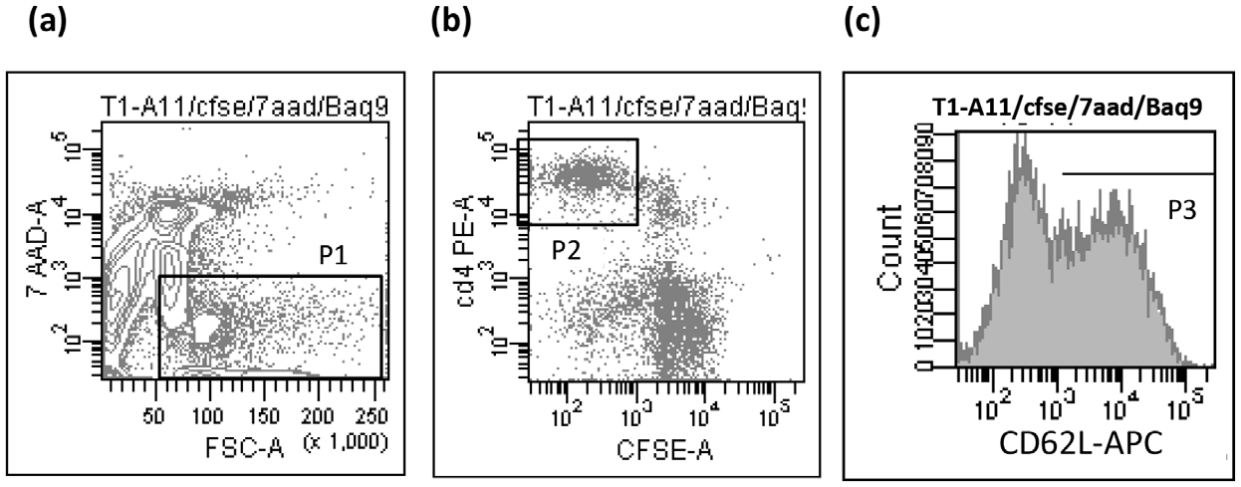
Both CD62L^+^CD4^+^ and CD62L^−^CD4^+^ T cells proliferate in response to *Mmm*SC stimulation *in vitro*. Results of a typical four-color flow cytometric analysis are shown for one vaccinated animal. Cells were loaded with CFSE and incubated for 9 days with inactivated *Mmm*SC before cell surface staining and analysis by flow cytometry as follows: (a) a first gate (P1) was used to exclude dead cells (7AAD+) and cell debris (FSC<50); (b) a second gate (P2) was set, among P1 gated cells, to delineate viable and proliferating CD4^+^ T cells (CFSElow or CFSE<1×10^3^); and (c), histograms were opened on gate P2 to obtain the percentage of CD62L^+^ cells among proliferating CD4^+^ T cells (i.e., P3).

We have shown previously that expression of CD62L among proliferating CD4^+^ T lymphocytes could discriminate between Tem and Tcm in *Mmm*SC presensitized animals [13]. Here, we have assessed the contribution of both populations in anamnestic T-cell responses of vaccinated cattle, focusing on animals that responded above nonvaccinated animals (i.e., 4 out of 5), to concentrate on vaccine-induced T-cell responses. We found that viable CD4^+^ T lymphocytes actively proliferating in response to *Mmm*SC comprised both CD62L^+^ and CD62L^−^ populations (Fig. 4). In general, the percentage of CD62L^+^ cells slightly exceeded the percentage of CD62L^−^ cells among proliferating CD4^+^ T lymphocytes (Table 1) whereas in non stimulated CD4 the ratio was 45±5%.

**Table 1:** Effect of *in vitro Mmm*SC stimulation on CD4^+^ T lymphocytes collected from cbpp-vaccinated cattle.

Animalnumber	% CD4^+^ T cells incultures stimulated^a^ with:		% proliferation^b^ among CD4^+^ T cells		% CD62L^+^ cells among proliferating CD4^+^ T cells
	NoAg	*Mmm*SC	NoAg	*Mmm*SC	
1	21.5±3	34±4	7±2	60±14	62±4
2	24.5±5	23.5±7	4±3	37±12	60±2
3	15±2	18±1	6±2	25±6	51±1
4	20±5	40±4	3±2	51±14	61±5

a pbmc were loaded with CFSE and incubated for 9 days with inactivated *Mmm*SC before cell surface staining and analysis by flow cytometry as described in material&methods. Results are expressed as mean (+/− SD) percentages of 2 experiments.

b Percentages of CFSElow cells within the gated CD4^+^ population.

**Figure 5:**
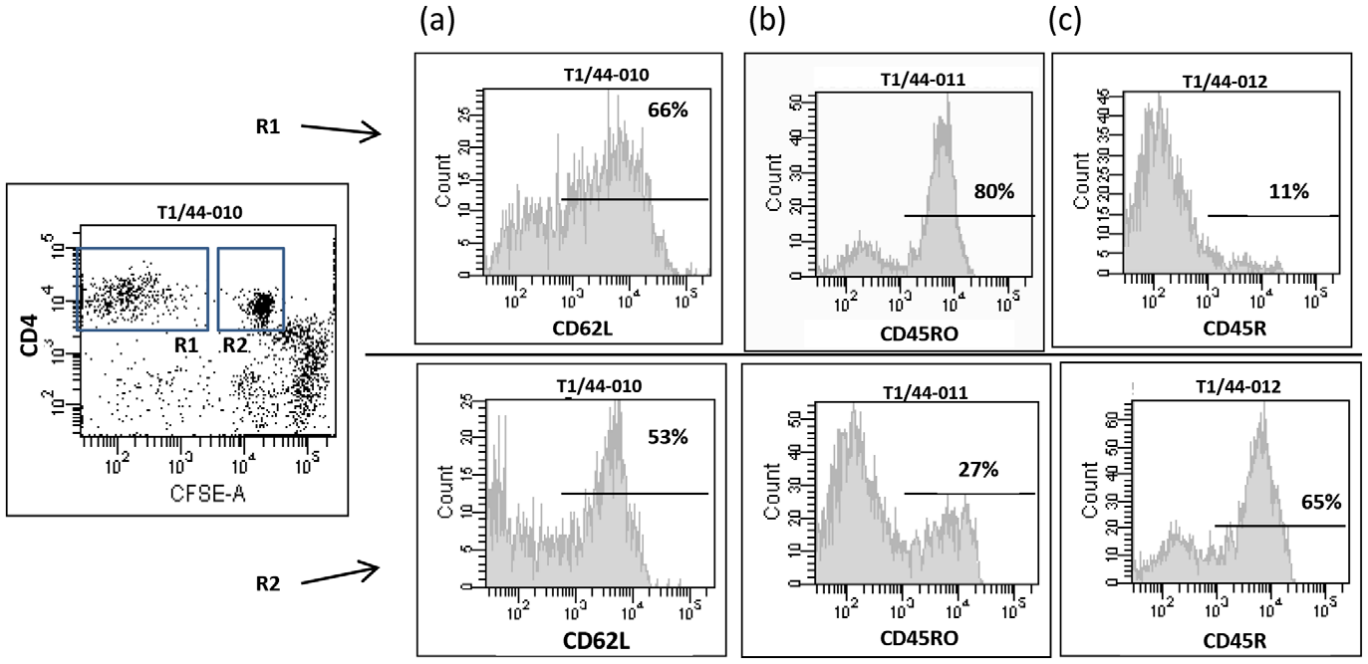
Cell surface expression of CD62L (a), and CD45RO (b), and CD45R (c) markers by proliferating (R1 gate) and non-proliferationg (R2 gate) CD4^+^ T cells stimulated with *Mmm*SC Ags. Percentages of positive cells within gated populations are shown on histograms.

Analysis of activation/memory markers indicated strong upregulation of CD45RO and down regulation of CD45R surface expression by proliferating CD4^+^ T lymphocytes (Fig. 5). It can be observed from Fig. 5 that, among proliferating CD4, the majority of CD62L^−^ and CD62L^+^ also followed that same expression pattern (i.e., upregulation of CD45RO and downregulation of CD45R) which is indicative of a memory phenoytpe.

## Discussion

The paucity of data on the immunogenicity of T1 vaccines against CBPP, and more specifically on CMI, has prompted this study. Our goal is to improve our understanding of protective immunity and facilitate the design of more effective CBPP vaccines that are urgently needed. Three African countries took part in the study to account for differences in national veterinary specificities and practices. This also allowed comparison of different vaccine strains and vaccination regimens.

In the first place, recall proliferation and IFN-γ production by PBMC, two markers of CMI, were chosen to assess the presence of anamnestic T-cell responses in vaccinated animals. Significant *Mmm*SC-induced recall proliferation and IFN-γ responses, both at individual and group levels, were detected exclusively in cattle that received three shots of T1 vaccines. These results confirm pioneer studies by Roberts and colleagues indicating that no recall activation of lymphocytes could be detected in cattle vaccinated two months previously in the tail tip with a single dose of the T1 vaccine [8]. A booster immunization one month later had little effect as shown in the present study. A possible explanation is that live T1 vaccines induce apoptosis in lymphocytes as has been shown *in vitro* for the T1/44 strain [14]. In our study, there was no obvious signs left of apoptosis in PBMC as indicated by uncompromised responses to the mitogen ConA (see Figure S1). Additional longitudinal studies are needed to fully explore the possibility that transient CMI is induced by a single shot of T1 vaccines.

Further characterization of CMI elicited by multiple shots of T1 vaccines underlined the determinant role of CD4^+^ T lymphocytes in recall *Mmm*SC-induced proliferation and IFN-γ responses. Although the contribution of other T-cell sub-types cannot be excluded, *Mmm*SC-induced responses *in vitro* were under the strict control of CD4^+^ T lymphocytes as shown by depletion studies. Moreover, CD4^+^ T-cell responders elicited by multiple injections of T1 vaccines comprised both Tem- and Tcm-like sub-types as suggested by CD62L surface expression. Interestingly, a predominant role for CD4^+^ T lymphocytes in CMI, including both Tem and Tcm, has also been reported for cattle that fully recovered from CBPP [13]. Full recovery from clinical disease is known to induce life-long protective immunity against CBPP [15], [16]. However, although qualitatively comparable, the magnitude of CMI was lower in T1-vaccinated cattle versus recovered cattle as indicated by several parameters (Table 1 and ref 13): 1) 2/5 v/s 10/10 animals had more than 50% of CFSElow cells among CD4^+^ T cells after *in vitro* stimulation; 2) 2/5 v/s 10/10 animals showed an increase in total CD4^+^ T cells among stimulated PBMC; 3) 0/5 v/s 10/10 animals showed above 70% CD62L^+^ cells among proliferating CD4^+^ T cells which is a marker for Tcm in our model [13]. This is consistent with the short-lived immunity afforded by T1 vaccines observed in the field which justifies costly annual booster immunizations campaigns [16].

Although not a final proof, this study further supports the idea that both CD4^+^ Tem and Tcm play a role in protective immunity since booster immunizations are associated with increased protection rate of T1 vaccines [17]. There is however a need for studies on CMI taking place in vaccinated/recovered cattle undergoing CBPP challenge to confirm the role of CD4^+^ T cells in protection. Moreover, the contribution of functionally different CD4^+^ effectors or Tem (i.e., Th1, Th2, Th17, Treg, polyfunctional CD4) will have to be clarified to understand the mechanisms underlying protective immunity [18]. This is especially true since severe CBPP may be associated with excessive cytokine production [19] and, thus, inappropriate immunization may exacerbate disease rather than protect. Additionally, this will allow identification of suitable correlates of protection that would facilitate future vaccine evaluation in cattle. Another important aspect of vaccine efficacy is long-term immunity particularly for CBPP to avoid expensive annual booster immunizations. Tcm mediate long-term immunity and have been associated with protection against chronic diseases [10, 20, 21, and 22], Moreover, in bovine tuberculosis, Tcm responses are detected long after Tem responses have waned and they are good correlates of vaccine-induced protection [23], [24].

Altogether, these results suggest that the immunogenicity of T1 vaccines is poor but that it can be increased by several booster immunizations. In that case, animals developed CMI that was qualitatively comparable to animals that have fully recovered from CBPP infections. However, quantitative analysis suggests that there is room for substantial increase in CMI, particularly in the Tcm compartment, even after three shots of T1 vaccines. This warrants further work to assess new boosting strategies other than homologous booster immunization. Our results also further support the idea that protection against CBPP is somehow associated with CD4^+^ Tem, the precise phenotype of which remains to be elucidated, as well as CD4^+^ Tcm. Therefore, markers of these responses have potential as predictors of vaccine efficacy and deserve further evaluation in future CBPP challenge studies.

## Supporting Information

Figure S1 Recall responses to the mitogen ConA measured by: i) proliferation among CD4^+^ T lymphocytes (a), ii) IFN-γ production measured by ELISA (b) and ELISPOT (c). Cells were collected from nonvaccinated (open circles) and vaccinated (closed circles) animals (n = 5) one month after single, double and triple vaccine inoculations. Results represent the net effect of ConA (i.e., stimulated cultures minus non-stimulated cultures).
(TIF)
