# Correction: Characterization of Anamnestic T-cell Responses Induced by Conventional Vaccines against Contagious Bovine Pleuropneumonia

**DOI:** 10.1371/annotation/1230acb5-cfc9-48e7-9a4b-569cec5cb05a

**Published:** 2013-08-16

**Authors:** Philippe Totte, Aboubakar Yaya, Amadou Sery, Hezron Wesonga, Abel Wade, Jan Naessens, Mamadou Niang, François Thiaucourt

The name and numbers of the grants from the European Union supervised by OAU/IBAR (Kenya) was incorrectly omitted from the Funding Statement. The name and numbers of the grants are: DCI-FOOD/2009/226-469 Vaccines for neglected animal diseases in Africa- VACNADA. 

