# Supplementary figures and images for: Characterization of Anamnestic T-cell Responses Induced by Conventional Vaccines against Contagious Bovine Pleuropneumonia
Source: PLoS One. 2013 Feb 28;8(2):e57509. doi: 10.1371/journal.pone.0057509 (PMC3585371; doi:10.1371/journal.pone.0057509)

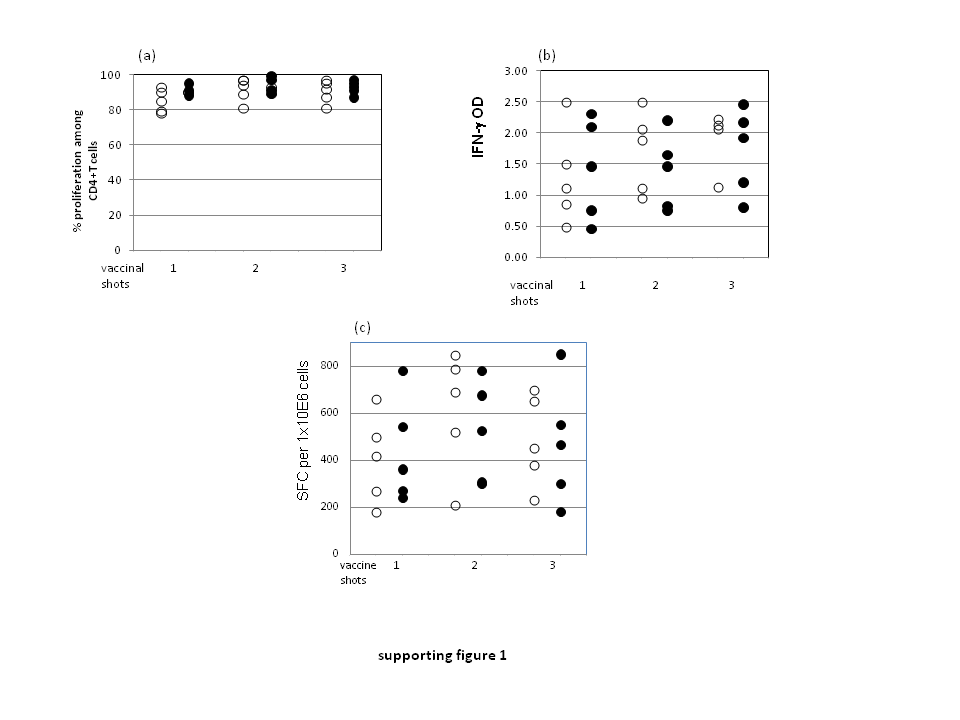

Supplement: Figure S1 — Recall responses to the mitogen ConA measured by: i) proliferation among CD4+ T lymphocytes (a), ii) IFN-γ production measured by ELISA (b) and ELISPOT (c). Cells were collected from nonvaccinated (open circles) and vaccinated (closed circles) animals (n = 5) one month after single, double and triple vaccine inoculations. Results represent the net effect of ConA (i.e., stimulated cultures minus non-stimulated cultures). (TIF) [file pone.0057509.s001.tif]
